# Supplementary material for: The impact of psychological theory on the treatment of Attention Deficit Hyperactivity Disorder (ADHD) in adults: A scoping review
Source: PLoS One. 2021 Dec 21;16(12):e0261247. doi: 10.1371/journal.pone.0261247 (PMC8691636; doi:10.1371/journal.pone.0261247)
Supplement: S1 Appendix — (DOCX) [file pone.0261247.s001.docx]

# Appendix 1

| **Table 1: Analysis of Cognitive Behavioural Treatments** | | | | | | | |
| --- | --- | --- | --- | --- | --- | --- | --- |
| **First Wave** | | | | | | | |
| **Author/Date** | **Intervention Style** | | **Characterisation** | | **Aims/Outcomes** | | **Theory** |
| Burgess et al 1997 | Individual | | Inability to maintain vigilance (attention deficit), distractibility, response inhibition, impulsivity | | Increasing the level of attending behaviour through contingent token reinforcement. Cognitive component unnecessary | | Wood (Brain injury) |
| Yankura & Dryden 1997 | Individual | | Cognitive deficits, response inhibition, Executive Function (EF) deficits, self-regulation, cross-temporal organisation of behaviour, diminished social effectiveness and adaptation | | Identify, challenge and replace beliefs and restructure disordered cognitions | | DSM IV, Barkley,  Douglas |
| Jordan 1998 | Individual | | EF deficits, self-regulation, disinhibition (Impulsivity), time blindness, aggressive, intrusive, unaware of nonverbal language of social signals, under aroused & stimulus seeking, distractable, poor listeners | | Recommends behavioural strategies for organisation, listening and scheduling | | DSM IV, Barkley |
| Carpentier 2004 | Individual + Coaching | | Neurobiological disorder of EF; impaired inhibition of stimulus response. Issues with planning, organisation, procrastination, restlessness | | Address secondary problems of underperformance, demoralisation and a negative self-image | | Barkley, DSM IV |
| **Second Wave** |  | |  | |  | |  |
| **Author/Date** | **Intervention Style** | | **Characterisation** | | **Aims/Outcomes** | | **Theory** |
| Goodwin & Corgiat 1992 | Individual | | Executive Function deficits | | Retrain or restimulate cognitive abilities, develop compensatory strategies, and/or modify the social and physical environments to promote optimal functioning | | Barkley, Wender |
| Weinstein 1994 | Individual + Coaching | | Hyperactivity, attention deficit, impulsivity, memory impairment. Biological-neurological disorder shaped by psychological and social factors. | | Multimodal approach recommended. Integrated cognitive and affective approach will increase awareness of behaviour and therefore control | | Barkley, Wender |
| Nadeau 1994 | Individual | | Executive Function deficits | | Retrain cognitive functions, develop internal/external compensatory strategies, restructure physical/social environment to maximise functioning | | Barkley, Brown |
| Jackson & Farrugia 1997 | Individual | | Inattention, overactivity, and impulsivity; develop negative attitudes due to lack of understanding; delaying gratification; Disregarding emotions of others, impairment of judgement & decision making; restlessness | | Controlling attention, breaking down tasks (sense of achievement = increasing motivation), self-awareness + self-monitoring + self-reinforcement, anger management training, problem solving | | DSM IV, Barkley,  Wender |
| Burt et al 1997 | Group | | Inattention, Hyperactivity, Impulsivity; inability to activate, mobilize, and sustain functions for non-interesting tasks. Poor organisation, self-regulation, low self-esteem, social performance defects, delay aversion, chronic procrastination, inaccurate self-observation | | Skills training in self advocacy, goal setting and achievement, problem-solving, stress management and cognitive restructuring | | DSM IV, Brown, Barkley |
| Wilens et al 1999 | Individual | | Inattention, distractibility, impulsivity; negative underlying beliefs, overreliance on dysfunctional strategies, and lack of development of normal adult coping skills | | Identification and elimination of maladaptive core beliefs and strategies, along with the strengthening of competing adaptive beliefs and strategies | | Barkley |
| Wiggins et al 1999 | Group | | Cognitive & physical restlessness, inattentiveness, distractibility, emotional lability, and impulsivity. | | behaviour change managed by goal setting, self-evaluation, reflection within group context | | DSM IV, Barkley, Wender |
| Young 1999 | Individual & Group | | Inattention, Hyperactivity, Impulsivity; Inadequate problem-solving skills, excessive attraction to immediate reward; poor time management and organisation; delay aversion; emotional lability; disorganisation; poor motivation | | Self-management although awareness of context is needed. Requires education about the disorder, cognitive-restructuring and reframing the past. | | DSM IV, Wood |
| McDermott 2000 | Individual | | Inattention, distractibility; Difficulty pulling away from interesting stimuli to stay focused. Reinforced negative beliefs can trigger excruciating emotions; | | Address cognition, behaviour and dysregulated. Training in self-evaluation, social skills, anger and impulse management, + self-instruction including coping skills and problem solving | | DSM IV, Barkley |
| Sohlberg & Mateer 2001 | Individual | | Memory impairment, problems in attention and concentration | | Environmental mods, attention training, self-regulatory strategies, external aids, psychosocial support. Self-management skills of orienting, pacing (energy), task switching | | Sohlberg & Mateer (brain injury) |
| Stevenson et al 2002 | Group + Coaching | | Cognitive impairments, skill deficits, dis-organized and chaotic working environments, and poor stress management. | | Designed to teach strategies to improve functioning in motivation, concentration, listening, impulsivity, organization, anger management and self-esteem | | Barkley, Wender |
| Wedlake 2002 | Individual | | Attention, impulse control, and hyperactivity. Distorted EF - impulse control, motivation, establishment of goals, planning, and personal assessment and management of behaviour and its consequences. | | Aim to improve working memory and enhance overall flexibility of cognitive processes by teaching problem solving, reasoning, and concept formation skills | | Barkley |
| Nadeau 2002 | Individual | | Memory difficulties, EF (disorganisation, time management, planning), social skills, self-regulation, low self esteem | | Neurocognitive Psychotherapy focusing on identifying strengths and weaknesses, cognitive reframing, acceptance, cognitive rehabilitation | | Barkley, DSM IV |
| Young 2002 | Individual | | Difficulty attending to tasks/activities and inhibiting impulses; poor concentration; poor sustained attention; impulsive; restless; lack of reflection/consideration; disorganised; self-regulation | | Psychoeducation, cognitive restructuring, reframing the past + behavioural techniques: graded task assignments, modeling, roleplay. Teach self-monitoring to regulate emotions - counterstrategy (distractive techniques) when overaroused. | | Barkley |
| Brooks 2002 | Individual | | Impulsivity, Low frustration tolerance, Moodiness, Disorganisation, Rigidity, Inflexibility, insatiability; self-regulation; lack of empathy; Negative mindset prompts self-defeating and counter-productive coping behaviours. | | Reinforce positive mindset and build resiliency. Solution Focused & problem-solving model for change. Encourage identification of personal strengths or "islands of competence" | | Barkley |
| Stevenson et al 2003 | Self-Help, Individual + Coaching | | Developmental, neurobiological condition, characterized by symptoms of inattention, hyper- activity and impulsivity | | Overcome attention and motivational difficulties, listening skills, organizational skills, impulse control techniques | | Barkley,  DSM IV |
| Ramsay & Rostain 2003 | Individual | | EF deficits. Developmentally inappropriate levels of impulsivity, inattention, and hyperactivity. Affective lability, hot temper, stress intolerance. restlessness, impatience, and agitation | | Develop greater sense of personal agency, to make informed decisions about life options, and to better manage the core and comorbid symptoms and associated problems. | | Barkley |
| Safren 2004 | Individual | | Neuropsychiatric disorder. Chronic cognitive and behavioural impairments, issues with attention, inhibition, and self-regulation | | Enhance organisation, planning and problem-solving skills; reduce distractibility via self-monitoring; eliminate dysfunctional thoughts | | Barkley |
| Ramsay & Rostain 2004 | Individual | | EF - impaired inhibition, planning, working memory, and cognitive processing speed. Developmental syndrome of impaired executive functioning significantly affecting relationships with environments. | | Correct maladaptive belief systems to reduce learned helplessness; gain sense of self–respect and resilience. | | Barkley |
| Safren et al 2005 | Individual | | impulsivity, attentional deficits, and disinhibition | | Increase compensatory strategies in organizing and planning, coping with distractibility, and enhancing optimal thinking strategies | | Barkley, Quay |
| Safren et al 2005 | Individual | | Distractibility, disorganisation, difficulty completing tasks, impulsivity. Impairments prevent effective coping, leading to underachievement and negative thoughts/beliefs = mood problems and task avoidance | | Maximise adaptive thinking (cognitive restructuring). Psychoeducation, organisation & planning skills; reducing distractibility | | DSM IV, Barkley |
| Young 2005 | Individual | | cognitive deficits of inattention and impulsivity | | Learn positive coping skills to apply in stressful situations: self-monitoring of maladaptive statements, behaviours and problem-solving | | DSM IV |
| Weiss et al 2006 | Individual | | Associated symptoms of low self- esteem, temper outbursts, mood dysregulation, reactivity, anxiety, poor motivation, and other symptoms that fall within the spectrum of internalizing disorders | | Management of comorbid mood disorders. Problem-focused therapy - education about ADHD, support to establish effective coping strategies | | Barkley, DSM IV |
| Murphy 2006 | Individual and Group | | verbal/behavioural impulsivity, disinhibition, | | Symptom reduction and minimisation of negative effects of disorder to improve QOL. Active and pragmatic treatment promotes best response. | | DSM IV TR, Barkley |
| Rostain & Ramsay 2006 | Individual | | inattention, impulsivity, and hyperactivity. Impaired executive function processes that affect self-regulation | | Develop and implement individualized adaptive coping strategies and identify and modify dysfunctional thoughts and beliefs | | DSM IV, Barkley, Wender |
| Gentile et al 2006 | Individual | | Difficulty starting tasks, variable attention to detail, difficulties with self-organization and prioritization, poor persistence in tasks requiring sustained mental effort. Impulsiveness, low frustration tolerance | | Assist with organization and planning, coping with distractibility, and cognitive restructuring. Learning skills to maximize adaptive thinking during stress | | DSM IV |
| Ramsay 2007 | Individual | | Developmental neurobehavioral syndrome of executive dysfunction -developmentally inappropriate levels of inattention, hyperactivity, and impulsivity. | | Cognitive modification addresses EF issues of self-management plus negative assumptions about capabilities, overgeneralized & interfering with possibilities of change. | | Barkley, Brown |
| Ramsay & Rostain 2007 | Individual | | Executive Function, Self-regulation - impaired behavioural inhibition, planning, working memory, and cognitive processing speed | | Therapist is active and directive to keep sessions focused. Cognitive modification fosters resilience. | | Barkley, Brown, Nigg,  DSM IV |
| Yovel & Safren 2007 | Individual | | Disorganization, forgetfulness, and distractibility, which are often associated with difficulty completing tasks | | Importance of homework adherence, in organizing and planning, coping with distractibility, and cognitive restructuring. | | DSM IV, Barkley |
| Young & Bramham 2007 | Individual and Group | | EF difficulties - origin of attention, memory and time management issues separate from impulsivity; attentional difficulties, poor motivation, poor organisational skills, impulsivity, anger management problems; | | Help individual change their environment, and develop psychological strategies for adaptive functioning in different environments. | | DSM IV, Barkley, Sonuga-Barke |
| Ross & Young 2007 | Group + Coaching | | Lifelong developmental disorder of EF dysfunction. Inadequate or delayed development of cognitive skills influences onset and persistence of offending behaviour. | | Teach problem solving skills, skills in emotion regulation, social skills and values, social perspective taking, the development of empathy, critical reasoning, negotiation skills and conflict resolution. | | DSM IV |
| Knight et al 2008 | Individual and Group | | Developmentally inappropriate levels of inattention, impulsivity, and hyperactivity. Inattentive symptoms of “sluggish cognitive tempo,” such as day-dreaming and processing deficits are common | | Recommends psychoeducation, skills training in organization, planning, and maximizing attention; and cognitive restructuring to maximize adaptive thinking and reduce distractions | | DSM IV |
| Ramsay & Rostain 2008 | Individual and Group | | Neurodevelopmental lifespan disorder of self-dysregulation and executive dysfunction extending across time and setting. | | Pharmacotherapy is single most effective treatment, but insufficient alone. Mentions psychosocial treatments needing more research for effectiveness as well as mechanism of change. | | Barkley |
| Ramsay & Rostain 2008 | Individual | | Neurobehavioral disorder affecting learning and cognition, behavioural, and emotional functioning consistent over time. Inattention, impulsivity, hyperactivity. Maladaptive compensatory strategies used to deal with negative beliefs | | Identify issues in behavioural practices stopped by negative beliefs. Exam stress generates "drift" away from coping skills - use cognitive restructuring to discover options | | Barkley, Brown |
| Toplak 2008 | Individual + Coaching | | Disorder of neural regulation and under arousal. Affected executive and cognitive processes; multiple pathways including executive and motivational; delay aversion; issues with reward schedules and extinction | | Training in self-instructions, problem-solving, self-reinforcement, and self-redirection to cope with errors. | | DSM IV TR, Barkley, Sonuga-Barke, Nigg |
| Virta et al 2008 | Group | | Developmental neurobiological disability characterized by deficits in attention and EF and/or symptoms of hyperactivity and impulsivity. | | Aim to improve participants’ skills in managing ADHD related problems and discuss problems with peers | | Brown, DSM IV |
| Young et al 2008 | Interviews | | Low self-esteem, hope- lessness, and underachievement | | Reframe past experiences in a positive way by encouraging them to learn from the past. Emphasise positive aspects of their disorder and/or their strengths (e.g., creativity) | | DSM IV, Barkley, Sonuga-Barke |
| Bramham & Young 2009 | Group | | Core symptoms of inattention, hyperactivity, and impulsivity; low frustration tolerance leading to angry outbursts; low self-esteem; mood disorders; impulse control, all-or-nothing thinking, and disorganization | | Improvement in psychoeducation, strategy development to improve self- esteem and self-efficacy | | DSM IV |
| Salakari et al 2010 | Group | | Deficits of organizing, prioritizing, and activating oneself to work; focusing, sustaining, and shifting attention to tasks; regulating alertness, sustaining effort, and processing speed; managing frustration and modulating emotions; utilizing working memory and accessing recall; monitoring and self-regulating action | | Themes and compensatory strategies for motivation, organization, attention, emotional regulation, memory, communication, impulsivity, psychiatric comorbidities, and self-esteem. | | Barkley, DSM IV |
| Virta et al 2010 | Individual | | EF deficits; Inattention, distractibility; Attentional dyscontrol (difficulty pulling away from interesting stimuli to stay focused) | | Compare focus on compensatory strategies, altering dysfunctional thoughts and attitudes, and improving metacognition | | DSM IV, Barkley, Brown |
| Knouse et al 2010 | Guidance | | Lags in the development of sustained attention and behavioural inhibition. Deficits in sustained attention, inhibitory control, working memory, and motivation | | Behavioural skills training to target the acquisition and especially the maintenance of compensatory skills | | Barkley, DSM IV TR, Nigg |
| Ramsay 2010 | Guidance | | Neurodevelopmental disorder - developmentally inappropriate levels of inattention, hyperactivity, and impulsivity. EF deficits, behavioural inhibition, working memory, affect regulation, reconstitution. | | Space for reflection and problem solving, reframe of sense of self and provide scaffolding through environmental engineering and EF Training; cognitive and behavioural modification to generate exposure to challenge negative beliefs. | | DSM IV TR, Barkley |
| Ramsay 2010 | Guidance | | Developmental syndrome of inappropriate impulsivity and overactivity coincides with attention and information processing difficulties. | | Cognitive remediation and cognitive rehabilitation to promote behaviour change and improve adaptive daily functioning through "self-control" therapeutic components | | Barkley, Nigg  Douglas |
| Safren et al 2010 | Individual | | Distractibility, disorganisation, difficulty following through on tasks, impulsivity. Impairments prevent effective coping | | Training in organising, planning and problem solving, skills to reduce distractibility, cognitive restructuring | | DSM IV TR |
| Newark & Stieglitz 2010 | Guidance | | Neuropsychological impairments: distractibility, disorganization, emotional instability, disinhibition, attentional problems, disorganized behaviour, insufficient self-regulation | | Coping techniques for independent goal achievement and self-efficacy. | | Barkley |
| Emilsson et al 2011 | Group and Individual + Coaching | | maladaptive personality and coping strategies which limits the internal resources available to the individual | | Aims to decrease impairment and improve social, problem solving, and organisational skills | | Barkley,  DSM IV |
| Fleming et al 2012 | Guidance | | Developmentally atypical levels of inattentive behaviour, hyperactive and impulsive behaviour; Self-regulation deficits in EF, working memory and a stronger aversion to delay in reward | | Enhancing motivation for change, training and practice of skills/coping strategies, cognitive restructuring of maladaptive thoughts, and accessing/ utilizing support | | Barkley, Sonuga-Barke, Sagvolden, Sergeant |
| Mongia & Hechtman 2012 | Guidance | | EF impairments: sustained attention, inhibitory control, working memory, and motivation; inattention, hyperactivity, and impulsivity | | Understanding and modifying cognitive distortions, self-evaluation, use of coping strategies, behavioural modification | | Barkley |
| Ramsay 2012 | Individual | | Developmental disorder of impaired EF: organizing/ executing behaviours across time to achieve future-focused objectives. | | Externalise EF and make treatment “sticky” using reminders | | Barkley |
| Weiss et al 2012 | Individual | | Self-perception of inadequacy and incompetence: poor self-esteem may remain embedded in the patient’s self-concept | | Problem Focused Therapy for improved functioning in EF skills, learning to self-reward, delegating, and optimising environment | | DSM IV |
| Young & Bramham 2012 | Guidance | | EF difficulties - origin of attention, memory and time management issues separate from impulsivity; | | Aim to practice until skills become automatic and routine – achievement is a strong reinforcer. | | Barkley, Sonuga-Barke |
| Mitchell et al 2013 | Individual | | Inattentive and hyperactive-impulsive. History of failure generates dysfunctional thoughts and beliefs, leading to negative emotional states, contributing to maladaptive compensatory strategies | | Cognitive restructuring of automatic thoughts leading to continued maladaptive behaviour and impairment. | | DSM IV TR, Barkley |
| Vidal-Estrada et al 2013 | Individual | | Developmental neurobiological disability causing functional impairment. Psychological variables contribute to maintenance of compensatory strategies and dysfunctional cognitions | | Provided info but not solutions for the following: symptoms recognition, disorder comprehension, causal and triggering factors, information about pharmacological and psychological treatment | | Barkley |
| Chandler 2013 | Systematic Review | | EF weakness: impulse inhibition, working memory, organisation, and planning skills; decreasing the ability to establish and follow through on reasonable goals and to manage distractions | | Organization and planning, coping with distractibility and cognitive restructuring; procrastination, anger management and communication skills | | Nigg, Barkley |
| Lindstedt et al 2013 | Individual | | EF deficits including: Planning, organising, fulfilling vocational goals, inattention, impulsivity, time management | | Cognitive Assistive Technology (CAT) for problem identification; personal and environmental resource identification; establishing individual goals. | | Barkley |
| Dittner et al 2014 | Individual | | EF deficits frequently associated with impairment, distress and psychiatric co-morbidity | | Collaborative focus on education, adaptations to environment and repetition of adaptive skills to compensate for executive dysfunction. | | Barkley |
| Knouse 2014 | Guidance | | Performance disorder - less attention, more intention; neurobiological disorder with core symptoms that impact EF; avoid aversive states by moving attention elsewhere | | Compensatory skills to ameliorate deficits - modifications to environment & behaviour Identify demotivating | | Barkley, Sonuga-Barke |
| Torrente 2014 | Interviews | | Neurobiological deficits in attention, executive function, and inhibitory control, failure, and underachievement in different domains of function | | Confirm hypotheses: dysfunctional cognitions would be positively correlated with depression or anxiety symptoms. Maladaptive coping strategies would correlate positively with dysfunctional cognition scales and/or emotional symptoms predicting poorer quality of life | | DSM IV, Barkley |
| Anastopolous et al 2015 | Group + Mentoring | | EF deficits resulting in chronic life-span condition associated with long term impairment in educational attainment, occupational status, and social relationships; increased risk for psychopathology and legal difficulties, deficits in self-regulation | | psychoeducation and skills training to help students cope more effectively with the executive functioning deficits inherent in ADHD | | Barkley |
| LaCount et al 2015 | Group | | Attention, impulse control, and activity-level modulation; Disorganization and inattention | | Skills training intervention including psychoeducation; organization, time management, and planning skills (OTMP); memory management; study skills and accommodations; increasing attention span; adaptive thinking; and mindfulness | | DSM V, Barkley |
| Knouse et al 2015 | Guidance | | Responsiveness to immediate rewards; delay aversion. Introduction of positively valanced cognitive avoidance - associated with both reductions in discomfort and increases in positive emotion | | Cognitive restructuring techniques - cognitive therapy modules targeting anxiolytic and depressogenic cognitions. Functional analysis of behaviour - identify how thoughts and behaviours function; avoid labelling cognitions as “irrational” or “distorted”. Implementation intentions - focus on changing the function of psychological events and relationship to them rather than changing events themselves | | Barkley, Sonuga-Barke |
| Knouse 2015 | Guidance | | Lifespan impairment including thinking patterns that consistently block the use of specific compensatory behavioural skills (e.g., organization and planning) | | CBT recommendations - Fit treatment to contours of semester; Measure skill use and functioning, not just symptoms; Consider power of the group; provide more frequent cues and support; Choose skills suited to the professional learner. Encourage self-testing as effective intervention in memory recall | | Barkley |
| Young et al 2015 | Individual + Mentoring | | Common neurodevelopmental disorder characterized by core symptoms of inattention, hyperactivity and/or impulsivity and associated with significant social, educational and occupational impairments | | Improve attentional control, memory, impulse control and planning; developing skilled thinking, problem identification, consequential thinking, managing conflict and making choices; managing feelings of anger and anxiety; recognition of the thoughts and feelings of others, empathy, negotiation skills, and conflict resolution; evaluating options and effective behavioural skills | | DSM IV, Barkley |
| Auclair et al 2016 | Systematic Review | | Difficulties with EF, specifically in sustaining attention, increased distractibility, a weak control over impulses, hyperactivity, emotional instability and disorganised behaviour | | Change problematic thoughts to create changes in emotions and behaviours. Understanding and modifying cognitive distortions, fostering the use of adaptive strategies, modifying behaviour and manage comorbid depressive moods, anxiety and self-esteem | | Barkley |
| Puente & Mitchell 2016 | Individual | | Developmental disorder with two core symptom clusters: inattention and hyperactivity-impulsivity. EF deficits in inhibition, attentional shifting, distractibility, organization, planning, and self-monitoring | | Targeting EF - Modules in psychoeducation, time management, overcoming emotional obstacles, activation and motivation, getting organised, and project planning | | Barkley, Nigg |
| Ramsay 2016 | Individual | | Deficits in EF and reward networks in the brain. Neurodevelopmental disorder characterized by clinically significant and developmentally inappropriate levels of inattention, hyperactivity, and/or impulsivity. Disorder of self-regulation; implicit motivation deficits | | Ability to “manufacture” enough motivation to start and persist on personally valued goals. Large task reframing to smaller tasks - progressive exposure intervention. EF/reward deficiency model useful for case formulation, treatment planning, and intervention. Self-regulation model framework moves beyond symptoms and promotes a richer understanding of ADHD | | Barkley |
| Strohmeier et al 2016 | Surveys | | EF deficits. Neurodevelopmental disorder characterized by developmentally inappropriate levels of inattention, impulsivity, and/or hyperactivity; Deficits in working memory, and organizing, sustaining, and completing tasks. Difficulty with self-regulation of emotion and motivation | | Focus on cognitive distortions; incorporate cognitive experience and enhance rationale for behavioural activation and exposure strategies; target task initiation cognitions, and promote management of emotional reactions; behavioural planning to increase likelihood of task implementation and follow through | | DSM V, Barkley |
| Wang et al 2016 | Individual | | Developmental impairments of cognitive functions, including inhibition, working memory, self- mobilization, organization, and time management characterized primarily by behavioural symptoms of inattention, hyperactivity, and impulsivity | | Psychoeducation and training in organization and planning; learning skills to reduce distractibility; and cognitive restructuring. Options for procrastination, relapse prevention and family support | | Brown |
| Young et al 2016 | Systematic review | | Neurodevelopmental condition - inattention, hyperactivity, and impulsivity. Impairments of cognitive functions: inhibition, working memory, self- mobilization, organization, and time management hinder learning or adapting coping strategies, resulting in continued difficulties. Potential history of failure or negative experiences may affect cognitions, affecting motivation, reducing likelihood of learning better coping | | Inattention, hyperactivity, and impulsivity underpinned by functional impairments but also include discouraging avoidance and procrastination; employing cognitive strategies to improve self-esteem through identifying, challenging, and replacing negative thought; behavioural skills training to develop effective coping strategies; psychoeducation to aid restoration of self- esteem | | DSM IV |
| In de Braek et al 2017 | Group + 1 individual | | Main symptoms are inattentiveness, hyperactivity, and impulsivity. Primarily a deficit in inhibitory processes involving executive function. Often suffer from mood swings and low self-esteem | | Teaching executive strategy will improve coping with cognitive failures and display fewer cognitive complaints. Training in goal management, defining problems, encoding, retrieval strategies, and self-monitoring | | Barkley |
| Pettersson et al 2017 | Self-help vs Self-help + group | | Hyperactivity, impulsivity, and inattentiveness affect everyday coping. Neuro- psychological deficits contribute to functional impairments by hindering acquisition and use of compensatory strategies, resulting in a history of failures and underachievement and dysfunctional beliefs; psychosocial and relationship problems, substance abuse, and problems with work performance and maintaining employment | | Teach self-directed compensatory techniques: behaviour analyses, time management, problem solving, organization and planning; Management of dysfunctional thinking (cognitive restructuring) and emotional distress (mindfulness and acceptance techniques) | | Barkley, DSM IV TR |
| Ramsay 2017 | Guidance | | Dimensional disorder. Neurodevelopmental problem of poor self- regulation. Difficulties developing, organizing, and sustaining behaviour across time toward a future focused goal. Executive function deficits, working memory difficulties, default-mode network = inattention and distractibility, dopamine system = motivation deficits, reward deficiency. Not associated with distorted cognitions but propensity for negative outlooks and excessive self-criticism are manifestation of deficient emotional self-regulation requiring cognitive intervention | | Consistent implementation of coping strategies is necessary for management. Behavioural and cognitive modification, EF coping skills, and ways to generalize these skills to various settings. | | Barkley, DSM V |
| Young et al 2017 | Group + mentoring | | Characterized by core symptoms of inattention, hyperactivity and/or impulsivity. Display impairments in their personal and social functioning (e.g. educational attainment, occupational difficulties and relationship problems | | Learning to improve attentional control, memory, impulse control and planning; Developing skilled thinking, problem identification, consequential thinking, managing conflict and making choices; Managing feelings of anger and anxiety; Recognition of the thoughts and feeling of others, empathy, negotiation skills and conflict resolution; evaluating options and effective behavioural skills. | | DSM V |
| Dittner et al 2018 | Individual | | Core symptoms contribute to the development of negative underlying beliefs (dysfunctional assumptions and core beliefs) and maladaptive coping strategies | | Emphasis on agenda setting, staying on-task during sessions, rehearsal of adaptive coping skills, problem-solving and revisiting history in linking current beliefs and behaviour with early life experiences | | Barkley |
| Jeavons et all 2018 | Guidance | | Neurodevelopmental condition with symptoms of inattention, hyperactivity, and impulsivity, cause significant functioning impairments. Impact on work and academic learning ranges from mild to severe | | Cognitive behavioural modification: coaching to increase everyday coping skills, and challenging unhelpful core beliefs affecting self-esteem and functioning. Make the most of strengths of the client. | | DSM V, Barkley |
| Knouse & Ramsay 2018 | Guidance | | Disorder of impaired self-regulation, chiefly executive dysfunction; difficulty turning intentions into actions and implementing component steps of valued endeavours | | Collaboratively addressing side effects to support skill development. Transparent informed-consent process, respect for patient autonomy, assessing, openly discussing, and validating emotional responses. Assist with setting reasonable expectations and managing maladaptive belief in failure by validating concerns and making relapse prevention plan | | Barkley |
| Luiu et al 2018 | Individual | | Neurodevelopment disorder characterized by inattention, impulsiveness, and hyperactivity (motor restlessness). Severe difficulties in organizing and planning activities, achieving goals and possess a very limited sense of time leading to poor time management. Difficulties inhibiting impulses and seek increased reward and sensation seeking behaviours | | Computerised phone app to support behaviour change through tailored messages to trigger both interest and motivation. Motivation fostered through positive rewards. Psychoeducation for Attention, planning, impulsivity, memory, and emotion regulation | | Sonuga-Barke |
| Solanto et al 2018 | Individual + group | | Executive self-regulation difficulties, inattention and adaptive impairment. “Temporal discounting”—i.e., the reduction in the perceived value of distant rewards | | Foster generalization and maintenance such that adaptive behaviours and cognitions become habitual and automatic. Change behaviour by imparting new skills and new habits related to the consistent use of those skills. Change cognitions considered adaptive internal speech or self-instruction to guide behaviour. Increase salience of distant rewards to activate, motivate, and sustain effortful behaviour toward long-term goals. Help create “rules” and develop adaptive cognitions to facilitate task initiation, completion, and planning | | Barkley |
| Huang et al 2018 | Group | | Developmental mental disorder. Multidimensional impairments including EF, academic performance, and occupational achievement as well as impulsivity, self-esteem, and life quality | | Improve core symptoms and reduce impairments in organization and planning, reducing distractibility, adaptive thinking, dealing with procrastination, building helpful relationship, and review. Booster sessions to consolidate skills and address relapse | | DSM IV, Barkley, Quay |
| Kooij et al 2019 | Guidance | | Problems with attention, impulsivity, and hyperactivity, emotional dysregulation and mind-wandering | | Psychoeducation, mutual recognition of lifetime symptoms and impairment, and reduction of stigma and self-blame. Coaching for sleep difficulties, anxiety, organisational skills, low self-esteem, depression, relationship issues, and financial problems. Support for low self-esteem or negative experiences due to failure or impulsive behaviour | | Barkley, DSM V |
| Holmefur et al 2019 | Group | | Executive dysfunction. Often have difficulties finishing tasks on time, and procrastination is common. Unrealistic understanding of time, often related to difficulties with regulation of emotions | | Improvements in time management, executive functioning, organisation, daily occupational satisfaction. Occupational therapy perspective of skill mastery using real tools, regulating emotions, learning that mistakes are integral to the process, and performing homework tasks | | Barkley, DSM V |
| Anastopolous et al 2020 | Group + mentoring | | Inattention, hyperactivity-impulsivity; self-regulation deficits, executive function deficits, Chronicity | | Intensive psychoeducation, EF (Goal setting, organisation, time management), CBT for maladaptive thinking, depression and anxiety, academic performance, social performance, treatment adherence | | Barkley, DSM V |
| Cherkasova et 2020 | Group + coaching | | Marked impairments in EF, long term goal attainment and impulse control, plus key areas of daily functioning, such as occupational, social, and family functioning, as well as psychiatric comorbidity | | Strategies for remediating functional deficits via behavioural compensation (organization, planning, time management strategies); Cognitive approaches to maladaptive thinking due to repeated failures contributing to maladaptive behaviours | | Barkley |
| Van der Oord et al 2020 | Individual | | Self-regulation, transition period, executive function (planning and motivational deficits, reward deficits, delay aversion) | | Improvement in academic functioning, comorbid symptoms and planning-related difficulties. Psychoeducation, personal goal setting, planning, study skills & distraction management, evaluation and repetition. | | DSM IV, Barkley |
| **Table 1: Analysis of Cognitive Behavioural Treatments** | | | | | | | |
| **Third Wave** | |  | |  |  |  | |
| **Author/Date** | | **Intervention Style** | | **Characterisation** | **Aims/Outcomes** | **Theory** | |
| Wasserstein & Lynn 2001 | | Individual | | Functional disconnection between the anterior and posterior higher cortical regions instead of a fixed dysfunction in either one. Disorders of attention, impulse control, motor activity, working memory and/or executive functions. Little demand for insight and self-awareness | Psychoeducation; Use of organizational systems and technologies. Works as prosthesis for EF deficits. Techniques grounded in stimulus-response paradigms and rely on models assuming an external locus of control for motivation or learning. | Barkley | |
| Hesslinger 2002 | | Group | | Dopaminergic hypoactivity, pre-frontal neuropathology. Attention deficit, emotional instability, disorganized behaviour and disinhibition. | Objective: Ability to control vs be controlled by ADHD - "Seize Control of ADHD". Psychoeducation with scientific knowledge regarding psychological and neurobiological aspects of attention and concentration. Behavioural analysis practiced in group sessions. Emotional analysis and regulation skills, impulse control skills via goal directed behaviour, stress tolerance skills and mindfulness skills | DSM IV, Wender | |
| Hesslinger 2004 | | Group | | attention deficit, hyperactivity incl. chronic inner restlessness, affective instability, being disorganized and impulse control deficit. Disorder of affect regulation, impulse control disorders, relationship disorders, addictive behaviours and lack of self-esteem – resembles personality disorder | Need for control (not “cure”) instead of being controlled. Psychoeducation around symptoms, deficits, and resources. Mindfulness skills to assist with impulsiveness and affective instability. Balance between validating acceptance of symptoms and the skills training for behavioural change | Wender | |
| Philipsen et al 2007 | | Group | | Persistent pattern of severely impaired attention and concentration, hyperactive and impulsive behaviour, emotional instability, restlessness, and disorganized behaviour. Deficits in emotion regulation, impulse control, sub- stance abuse, low self-esteem, and disturbed interpersonal relationships | Objective: Ability to control vs be controlled by ADHD. Psychoeducation, scientific knowledge regarding psychological and neurobiological aspects of attention and concentration, disorganisation strategies, behavioural analysis skills, emotional analysis skills, exercises in impulse control, stress management techniques, symptoms of dependency, relationships and self-esteem and planning for the future. | Barkley, DSM IV | |
| Solanto et al 2008 | | Group | | Inattention/memory problems, self-mobilization, organization, time management, and sustained effort. Poor focused and sustained attention, disorganization, and forgetfulness. Problems with time management represent the convergence of dysfunctions in time perception, sustained attention, resistance to distraction, and planning | Time management, organizational, and planning skills to improve immediate response rate and maintenance of gains; Sufficiently intensive and extensive to bring about enduring change in behaviour-development of new skills, behavioural repertoires, and adaptive cognitions; practical and easily incorporated into daily life becoming habitual and automatic and no longer dependent on active executive or decision-making functions | Barkley, Nigg | |
| Jacob et al 2008 | | Group | | Attention disorder, motor hyperactivity, impulsiveness, affectability, emotionally over-responsiveness, disorder of affect control, disorganised behaviour | Control of ADHD Symptoms. Homework is integral. Recommends behavioural modifications to work, leisure and social interactions. | Wender | |
| Baijal & Gupta 2008 | | Guidance | | Poor sustained attention, impulsiveness, and hyperactivity. Response inhibition, error monitoring, attentional disengagement, decision-making processes, and emotion regulation. problems in selective attention functions that are necessary to perform a given task | Reduce symptoms of stress, anxiety, and depression. Stylized attentional deployment, reframing of cognitive context, and emotional regulation | DSM III, Barkley | |
| Zylowska et al 2008 | | Group | | Behavioural disorder associated with cognitive impairments and brain alterations (structural and functional); Cognitive deficits in EF, including attention, working memory, inhibition, inattention, and impulsivity. Secondary impairments of stress, anxiety, and depression. Emotional dysregulation | Mindfulness - mental training to regulate attention and brain function. Improve self-regulation of attention and emotion. Rehabilitate aspects of attention and executive function | Barkley, Nigg, DSM IV | |
| Zylowska et al 2009 | | Group | | Behaviourally defined condition characterized by a clustering of symptoms of inattention and/or hyperactivity and. Comorbid disorders of learning and social–emotional development, including dyslexia, executive function deficits, and social problems | Psychoeduction including emotion regulation difficulties and emotional function and acceptance. Describe as “neurobiological difference” representing one extreme on a spectrum of functioning to destigmatise and re-frame impairment. Encourage taking responsibility as in coaching. Encourage persistence. Development of attention awareness and non-judgemental meta-awareness leading to discernment, choice and mindful action | Barkley, DSM IV | |
| Solanto 2010 | | Group | | Executive dysfunction; deficit in inhibitory control, arousal and activation; Inattentive - distractability, difficulty tracking time, initiating tasks lacking interest, losing items, unfinished projects; Hyperactive-Impulsive - intolerance of delay, insufficient planning, motor overactivity/restlessnes | explicit skills training, strategies to compensate for EF difficulties, enhance reinforcement, identifying/resisting emotional distracters | Barkley, Sergeant, Nigg, Sonuga-Barke, DSM IV | |
| Solanto et al 2010 | | Group | | Difficulties in the attentional domain more prevalent than in the hyperactive-impulsive domain; impulsivity, social behaviour, and mood control are common only to a subset of patients and require a different intervention format | Provide contingent self-reward (e.g., for completing an aversive task); Dismantle complex tasks into manageable parts; and sustain motivation toward distant goals by visualizing long-term rewards. Address anxious and depressive cognitions. | Barkley | |
| Philipsen et al 2010 | | Group | | Affective lability (extreme mood swings), hot temper, disorganization, stress intolerance and impulsivity | Psychoeducation, mindfulness training, organization skills (‘‘chaos and control’’); self-management of behaviour analysis; emotion regulation, depression and medication; impulse control, stress management; substance abuse and dependency; Relationships and self-respect | DSM IV, Wender | |
| Hirvikoski et al 2011 | | Group | | Deficits in executive function - impulse inhibition and working memory, as well as organization and planning skills | Ability “to control ADHD rather than to be controlled by ADHD”. Psychoeducation, scientific knowledge regarding cognitive dysfunctions and introduction of mindfulness skills; behavioural analysis skills, emotional regulation skills, exercises in impulse control and positives of impulsivity; stress management techniques; organisation and planning strategies; symptoms of dependency, relationships and self-esteem and planning for the future. | Barkley, DSM IV, Wender | |
| Fleming et al 2013 | | Group + coaching | | Executive function deficits, working memory; characterized by developmentally atypical levels of inattentive behaviour, hyperactive and impulsive behaviour, or both | Skills training included psychoeducation and EF, mindfulness practice, organization, and planning, structuring environment, managing daily life rhythms, and emotion regulation. Daily tracking essential. | Barkley, DSM IV TR | |
| Mitchell et al 2013 | | Group | | Inattentive and hyperactive-impulsive; EF deficits and emotion dysregulation; Emotional impulsivity and self-regulation. | Orient attention purposefully to the present moment and approach present moment experience with curiosity, openness, and acceptance | Barkley, DSM IV TR | |
| Schoenberg et al 2014 | | Group | | Impairments in performance monitoring or flexible and adaptive goal-directed cognition and behaviour, encompassing error processing and conflict monitoring subsystems which update and modify subsequent response. Likely dopaminergic system activity within interplay between cognitive and affective dynamics in error processing | Improve attention and self-regulation through attention regulation, emotion regulation, somatic awareness, distancing from a self-focused perspective. | Barkley | |
| Philipsen et al 2015 | | Group | | Mental disorder associated with numerous co-  morbid disorders and negative psychosocial consequences | Aim of our study was to demonstrate the efficacy of highly structured behavioural GPT compared with less specific treatment | DSM IV, Wender | |
| Bueno et al 2015 | | Group | | inattention, impulsivity, hyperactivity, and affective problems, decreased mood and arousal, and low motivation due to issues with cognitive control and emotional regulation | Cognitive training involving mindfulness to develop strategies that improve attention, affective self-regulation, and well- being and quality of life | Barkley | |
| Janssen et al 2015 | | Group | | Common neurodevelopmental disorder; inattention and/or hyperactivity-impulsivity; executive function deficits, attention regulation | Paying attention in a particular way: on purpose, in the present moment, and non-judgmentally. Strengthen attention regulation and improve executive function and emotion regulation | DSM IV | |
| Mitchell et al 2015 | | Individual | | Developmental condition - executive functioning, and emotion regulation; mind wandering - default mode network implicated in poorer attentional regulation; emotional impulsivity | improvement in mind wandering, emotional regulation, resisting impulsive urges to act out; improvement in self- reported inattentive, hyperactive-impulsive, depressive, and anxiety | Barkley, DSM V | |
| Hepark et al 2015 | | Group | | Executive function deficits - inter-related higher-order abilities involved in self-regulatory functions that organize, direct, and manage cognitive activities, emotional responses, and overt behaviour | Self-regulation of attention so that it is maintained on immediate experience with an attitude of curiosity, openness, and acceptance | Barkley, DSM IV TR | |
| Ramsay & Rostain 2016 | | Guidance | | Neurodevelopmental EF disorder interfering with development and consistent use of effective coping skills. “Implementation” disorder: difficulties organizing, executing, and sustaining plans across time to accomplish personally relevant endeavours. Self-regulation and motivational deficits. Default-mode network (DMN) suppression issues and dopamine deficiency. Escape behaviours driven by anticipation of discomfort vs actual feelings | Address EF: motivational and performance deficits and coexisting issues related to mood, anxiety, and self-esteem. Help consistently implement coping strategies in daily lives to compensate for self-regulation difficulties and improve functioning | Barkley, Brown, DSM V | |
| Bachman et al 2016 | | Guidance | | EF deficits: inattention, impulsivity, and hyperactivity. Abnormal neuronal activity in dorsofrontostriatal, orbitofrontostriatal, and frontocerebellar circuits and abnormal functional connectivity in the DMN. Altered connectivity within and among several neural networks, rather than abnormal functioning of discrete, isolated brain regions | Modification of underlying, dysfunctional neuronal systems, because of concrete, intense, and repetitive stimulation induced by exposure sessions. Improve attention control and emotion regulation by strengthening the function of brain regions. Deliberately target neuronal mechanisms associated with psychiatric disorders, similar to pharmacological or surgical treatments. | Nigg | |
| Cairncross & Miller 2016 | | Systematic review | | Neurobehavioral disorder: developmentally inappropriate, pervasive, and persistent symptoms of inattention and/or hyper- activity/ impulsivity. Difficulty sustaining attention in tasks that require self- regulation and task- or goal-directed persistence. Inability to inhibit and control external and internal stimuli that interfere with EF for self-regulated persistence. Overactivity and poor impulse control. | Provide externally represented information to guide behaviour,and enhance motivation. Training to self-instruct on task approach, self-evaluate, and provide self- reinforcement upon completion of a task. Learn to identify and respond to negative thoughts. Decrease hyperactivity/impulsivity and increase attentional capacity | Barkley, DSM V | |
| Aadil et al 2017 | | Systematic review | | Neurobehavioral disorder with high levels of inattention, impulsiveness, and hyperactivity. Difficulty prioritizing, organizing, and completing tasks, variable attention to detail, hyperactivity, and reduced impulse control | Improve task completion, self-regulation, and impulse control. Increase awareness, identify, and respond to physiological and psychological processes involved in maladaptive behaviours and emotions | Barkley, DSM V | |
| Edel et al 2017 | | Group | | Inattention, hyperactivity, and impulsivity | Decrease in executive functioning, and emotion dysregulation symptoms and increase in self-efficacy and mindfulness skills | Barkley | |
| Lee et al 2017 | | Systematic review | | Neurobiological disorder described by symptoms of inattention, hyperactivity and/or impulsivity | Develop skill of conflict attention. Observing and attending to the moment-to- moment changes of thoughts, feelings, and sensations. Maintain curiosity about mind wandering and open to the present moment. | DSM IV | |
| Hoxhaj et al 2018 | | Group | | Highly impairing mental disorder of executive functioning and emotional dysregulation with comorbid depression and anxiety | Focus attention on the present moment and develop a non-evaluative attitude. Improve self-regulation of attention, practice focusing of attention, and facilitate a constructive approach to dealing with negative feelings and judgemental cognitive processes preventing an open and accepting attitude | Barkley | |
| Bachman et al 2018 | | Systematic review | | Neurodevelopmental disorder characterised by inattention, hyperactivity, impulsivity and impairments in working memory, emotion regulation and inhibition control | Improve working memory capacity by strengthening the function of the frontal and parietal brain areas. Increasing awareness of internal and external experiences of the present moment and respond more adaptively to the physiological and psychological processes involved in psychiatric disorder. Improve attention, emotion regulation and quality of life | DSM V, Nigg | |
| Gu et al 2018 | | Individual | | Functional deficits related to decreases in attention, self-monitoring, inattention, impulsivity, executive functioning, and mood | Sustained attention training, emotion control, somatic awareness, non-judgmental awareness, curiosity, and acceptance of the “here-and-now,” distancing from a self-focused perspective, openness to present experience. positive effects on attention, mood, self-regulation, executive function, behaviour problems, and quality of life | Barkley, Sonuga-Barke, DSM IV | |
| Janssen et al 2018 | | Group | | Neurodevelopmental disorder with issues in executive functioning and attention regulation | improved attention regulation, enhanced brain activity and altered attention-related brain areas. strengthen functioning in brain regions that underlie neuropsychological deficits. Reduce symptoms and improve executive functioning, mindfulness skills, self-compassion, positive mental health and general functioning | DSM IV TR | |
| Lam et al 2019 | | Group | | Hyperactivity/impulsivity, inattention and memory problems, emotional lability; Issues with self-awareness and self-control | Long term efficacy for treatment of core symptoms and depression based on cognitive group psychotherapy | DSM IV | |
| Xue et al 2019 | | Systematic review | | Neurodevelopmental disorder characterized by core symptoms of inattention and hyperactivity/impulsivity, excessive motor activity, and difficulty in maintaining attention | Improve self-regulation abilities and quality of life. Emphasize non-judgemental observant and nonreactive attitude toward thoughts, emotions, and body state in the present moment. Improvement of attentional regulation involving executive functioning processes | DSM V | |
| Ramsay 2020 | | Guidance | | Neurodevelopmental disorder of impaired self-regulation. Hyperactivity, impulsivity and inattention at developmentally inappropriate levels that cause impairment. EF construct defined as self-directed behaviours to organise goal-directed plans, implement and sustain action over time, and achieve personally salient outcomes for which there is deferred outcome or reward | Accurate self-monitoring of cognitive distortions by assessment of reflexive, automatic “hot” cognitions and reflective “cool” processing; cognitive modification skills; de-energising intrusive thoughts with cognitive defusion; learning adaptive cognitive reframing skills. Reinforce progress by normalising processes of change and highlighting personal competence, strengths and aptitudes. | Barkley, Nigg, Brown, Sonuga-Barke | |

| **Table 2: Analysis of Coaching Treatments** | | | | |
| --- | --- | --- | --- | --- |
| **Author/Date** | **Intervention Style** | **Characterisation** | **Aims/Outcomes** | **Theory** |
| Hallowell & Ratey 1994 | Guidance | Neurological syndrome of attention inconsistency characterised by sense of underachievement - not meeting goals; difficulty getting organised; chronic procrastination; multiple incomplete projects; thoughtless comments; search for high stimulation; intolerance of boredom; distractibility; difficulty focusing attention; creative, intuitive, intelligent; impatient; impulsive; anxious; insecure; mood swings; restless; inaccurate self-observation | Coach as active, encouraging role of therapist. Not psychoanalytically oriented but acts as a structuring force for patient. Remind patient of goals and objectives in encouraging way to help stay on track. Encourages development of insight | Wender,  Douglas, Barkley |
| Jaksa & Ratey 1999 | Guidance | Problem is not one of desire or motivation but of follow-through and achievement. Individuals live in the moment, respond to the immediate, have difficulty anticipating and looking ahead, or simply forget what the behavioural goals were from a few days ago | Behaviour therapies limited. Mutually agree on approach. Take active role in terms of offering suggestions, goal completion paramount along with skill building. Feelings may involve frustrations, fears of failure, avoidance behaviour, and loss of confidence - address motivation influence on behaviour and goal achievement. Experience of overcoming past obstacles may produce heightened self-esteem and reduction in stress, anxiety, and worry. | DSM IV |
| Quinn et al 2000 | Guidance | Executive function difficulties - Difficulty focusing and sustaining attention; distracted by motivating/stimulating activities; weaknesses in problem solving skills and exerting self-control | Rewards and consequences; environmental engineering; setting goals and planning; self-observation; student as expert | Barkley |
| Ratey 2002 | Guidance | Executive function difficulties - under aroused attention; negative emotional overwhelm; lack of motivation; under functioning in planning, prioritising, attending to details, following through | Time management, prioritising tasks/directing attention, self-monitoring. Focus on what, how, when - never why. Not restricted by location or set model of contact/frequency. Help with readiness for change, goal support focus, negative self-talk, forgetting pain of procrastination (discomfort aversion); self-observation, event processing. Ask non-judgemental questions, provide feedback, establish structured routines, hold client accountable. | No primary citation |
| Nadau 2002 | Guidance | Hyperactivity/Impulsivity, but calls to adjust criteria for women - underachieved potential, time management/organisational issues | Clarify goals, set priorities, build habits, complete complex multi-step tasks. Focus of work is daily life management vs emotional | DSM IV, Barkley |
| Goldstein 2005 | Guidance | Significant impairment and psychological dysfunction requiring supportive treatment | Editorial criticism of lack of evidence base for effectiveness of coaching. Recommends peer reviewed research | No primary citation |
| Swartz 2005 | Individual | Deficits in executive functioning; inattention, impulsivity, disorganization, and a lack of self-regulation | Independently develop internal and external structures. Support long term goals and weekly objectives using rewards and consequences. Improve procrastination, lack of concentration, ineffective self-regulation, poor planning, anxiety, social incompetence, and time management. | Barkley |
| Reaser 2008 | Individual | EF deficits; inattention, impulsivity, and hyperactivity; motivational impairments are core–preference for easy work, less enjoyment of learning, less persistence, and greater reliance on external vs internal standards to judge performance. Deficiencies in the regulation of behaviour to situational demands, self-directed instruction, self-regulation of arousal to environmental demands, and in rule-governed behaviour. | Through continuous reinforcement, positive behaviours and self-efficacy will be strengthened, while negative habits will weaken. Punitive consequences recommended. | DSM IV TR, Barkley, Quay |
| Weyand & DuPaul 2008 | Guidance | Inability to sustain attention, impulsivity, and hyperactivity, impaired organizational skills, study skill deficits, deficits in executive function, lower quality of life, and issues with motivation, anxiety, information processing | Controlled investigations of pharmacotherapy, psychosocial, and educational interventions needed, particularly about impact on academic, social, and psychological functioning beyond symptom reduction. | DSM IV TR, Barkley |
| Parker & Boutelle 2009 | Qualitative | Pervasive impairment in the self-regulation of behaviour and affect due to developmental difficulties with executive function | Support students in developing systems and strategies. Help learn to take action while viewing as creative and resourceful. Promote self-determination of students with disabilities | Barkley |
| Assheton 2009 | Guidance | Disorganisation and procrastination to life-limiting, significant impairments in EF. Struggle to stay on track during a conversation; short-term/working memory difficulties – external prompts needed for the sometimes sluggish, and often distractible, ADHD brain. | Provide support, structure and accountability to improve performance and supports growth and change. Collaborative, action oriented, enhancing quality of life. Explore strengths, talents, tools and new learning to improve problem-solving and decision-making, and increase self-awareness and personal empowerment. | Barkley |
| Hugh & Ackerman 2010 | Qualitative | Lack of control over EF: procrastination (starting), hyperactivity (stopping), distraction (inhibiting), disorganisation, and switching between complex tasks. | Help design systems that are easy and natural instead of teaching optimal organisation schemes. | Barkley, DSM IV |
| Kubik 2010 | Qualitative | Disability with deficits of inhibition, self-regulation of emotions, and working memory, affecting every aspect of life | Move from a negative self-concept to the reality of capability. Discussion of blame, excuses, or defensive behaviour prompts insight to create behavioural controls strategy. Stress need for personal discipline. Daily check-ins required to encourage consistency. | Barkley |
| Murphy et al 2010 | Guidance | Executive Functioning - behavioural inhibition, working memory, and self-regulation of affect–motivation–arousal complex. Extreme emotional swings and disruptive experiences. | Identify strategies that circumvent defects in EF. Facilitate self-initiated change. Recommends hierarchical goal achievement process. | Barkley |
| Ramsay 2010 | Guidance | Developmental syndrome where symptoms may be evident, but impairment may not arise until normative environmental demands exceed EF capabilities. Capacity for behavioural inhibition and foresight foundational for self-control and regulation. Developmentally inappropriate impulsivity and overactivity coincides with attention and information processing difficulties. | Overview of non-medication treatments available. Environment framed as becoming progressively "more challenging" and therefore disabling. | Barkley, Douglas, Nigg |
| Farmer 2011 | Individual | Neurobehavioral disorder which is characterized by difficulties with inattention, hyperactivity, and/or impulsivity. Struggle with EF tasks: planning and self-monitoring, difficulties with organization, self-regulation, and procrastination | Encourage reflective thinking using guided cognitive instruction to guide and identify appropriate strategies for goal achievement. Procedural cognitive strategy instruction to teach skills, provide scaffolded practice aiming for internalisation and adaptive application | DSM IV TR |
| Privatt et al 2011 | Individual | Procrastination, lack of concentration, ineffective self-regulation, poor planning, anxiety, social incompetence, or time management | Cognitive restructuring changes occur through behavioural interventions: questioning, problem solving, modelling, and practicing. Focus on organizational skills, time-management, goal setting, and specific study skills | Barkley |
| Parker et al 2011 | Individual | Executive functioning impairments, attentional impairments, overwhelmed by new academic and organizational demands as they transition to post- secondary campuses, providing less external structure compared to high school and home settings | Model effective EF. Listen without judgment, affirm feelings. Support self-determined goal achievement and reduce levels of daily anxiety and stress. Help make goals more specific, develop more realistic plans for achievement, notice and report on ensuing barriers or successes, and be accountable for their efforts to act on their plans. | Barkley,  Brown |
| Bowles 2012 | Guidance | Pervasive and abnormal levels of hyperactivity, impulsivity, and/or inattention; difficulty focusing, meeting deadlines, following tasks through and sustaining effort and motivation for boring or irrelevant tasks. Easily sidetracked, forgetful, inconsistent work and school performance, disorganized, failing to plan ahead, mood swings and a quick temper, and difficulties in social relationships and marriages. Poor management of finances, low work productivity, irresponsible behaviour, and impaired social skills | Address the daily challenges of living and learn practical skills necessary for change. Identify barriers to attaining goals and generate specific, behavioural plan. Classical and operant procedures used. Provide motivation, and organization | Douglas, Barkley, DSM IV TR |
| Field et al 2013 | Individual | Executive Function, self-regulation for organizing, directing, and managing other cognitive activities, emotional responses, and overt behaviours. Executive functioning impairment believed to be the underlying cause | Help assess environments, identify needs, set goals, offer suggestions and guidance, and hold accountability. Set set structure, provide support, and help implement strategies for skill building. | Brown |
| Parker et al 2013 | Qualitative | Impaired executive functioning underlying cause impacting self-regulatory abilities -mechanisms for organizing, directing, and managing other cognitive activities, emotional responses, and overt behaviours | Ask questions to prompt reflection and planning. Facilitate ability to clarify goals and create realistic plans for achieving them. | Barkley, Brown |
| Wright 2014 | Guidance | Designated mental health disorder - genetically related, neurologically brain-based difference. Research shows EF plays key role. Psychological and physiological challenges of anxiety, depression, sleep disorders co-occur. | Seeking evolutionary change, strengthening identity and values, bringing dreams/goals into reality - inspiring action. Design prosthetic environments for scaffolding at key points of performance; conscious awareness of resources and abilities | Barkley, DSM IV |
| Richman et al 2014 | Individual | Significant difficulties with organization, time management, goal setting, and stress management; Deficiencies in problem solving, decision making, and inhibitory functions | Collaborative engagement, active listening and promotion of self-discovery and action by posing meaningful questions designed to trigger deeper critical reflection. Accountability for follow through on goals, plans, and commitments in a non-judgmental manner | Barkley |
| Prevatt et al 2015 | Individual | EF deficits - procrastination, lack of concentration, ineffective self-regulation, poor planning, anxiety, social incompetence, or time management. Limited capacity for EF and self- control | Compensate for EF impairment. Activities to replenish EF “fuel tank” important – frequent breaks, regular exercise. Cognitive changes through behavioural interventions – questioning, problem solving, modelling, and practicing. | Brown, Barkley |
| Farmer et al 2015 | Individual | Lack the skills and strategies (e.g., self-regulation, organization, study skills) needed for college settings | Guided cognitive instruction to develop goals, plans to achieve the goals, and plans to monitor the progress on goal achievement. Use learning and signature character strengths to meet weekly goals, focusing on strengths vs deficits. | No primary citation |
| Prevatt 2016 | Guidance | Normal developmental challenges amplified due to inherent deficits in self-regulation. Need improvement in executive functioning skills - time management, prioritization, developing realistic plans, activating, and sustaining effort over time, remembering goals, and regulating emotional reactions. | Pragmatic, behavioural, results and action-oriented approach targets planning, time management, goal setting, organization, and problem solving. Address academic, vocational, and interpersonal life difficulties due to deficits in EF. | Barkley |
| Schrevel et al 2016 | Qualitative | Inattention, hyperactivity, and impulsivity | Reinforce and utilize individual innate competences to achieve goals. Focus on collaboration and client uniqueness | DSM IV TR, DSM V |
| Bomar 2017 | Individual | Developmental difficulties with executive functions and cognitive functions. Lower emotional intelligence and academic self-efficacy | Develop skills, strategies and beliefs essential to overcoming the challenges of EF difficulties. Support independent learning by constructing personalized strategies. Learn to monitor behaviour and transfer responsibility of change to the student | Brown |
| DuPaul et al 2017 | Quantitative | Difficulties are presumably due not only to inattentive and/or hyperactive–impulsive symptoms, but also to a lack of adequate preparation in academic, organization, time management, and study skills | Foster student autonomy, self-determination, and metacognitive awareness. Nondirective approach fostering meta-cognitive thinking | Barkley, DSM V |
| Prevatt et al 2017 | Individual | Exhibit poorer EF skills. forgetfulness, poor motivation, lack of ability, avoidance, and poor time management. Working memory deficits - inability to remain focused on a future goal, and relate to common occurrence of task switching | CBT approach - planning, time management, goal setting, organization, and problem solving. Use reward/consequence to support goal achievement | Barkley, Brown |
| Singley 2017 | Individual | Poor organizational and time management skills, poor study skills, and poor social skills. challenges in executive functioning skills | Reflection, planning, goal setting. Use feedback, relationship building, insight, and competency development - behavioural techniques and strategies to complete goals in a systematic way. | Barkley |
| Ahmann et al 2018 | Descriptive review | EF challenges. Difficulty managing variable course schedules, expanded autonomy, decreased structure, an increased number of distractions, and reduced social supports, including a reduction in parental supervision and support | Egalitarian and nonclinical - personal-development orientation. Increased accessibility and accountability. Focus on skill acquisition and implementation. Flexible non-traditional meeting structure. | Barkley |
| Parker et al 2018 | Guidance | Self-regulation impairments; difficulties with working memory, activation, and motivation; experience difficulties setting and achieving goals due to executive dysfunction challenges; delay in prefrontal cortex development EF occurs. | Client is creative, resourceful, whole, and co-active agent. Powerful, brief, non-therapeutic and open-ended questions designed to elicit use of EF skills to recall the past, identify options, and make realistic plans for goals. Accountability plan key in between sessions - reinforcement cycle is integral | Barkley |
| Goudreau & Knight 2018 | Individual | Self-regulation and EF skills struggle including time management, organization, prioritizing, focus and attention, planning, motivation, and impulsivity. Difficulty paying attention to information when not interesting, remembering daily medications, organizing daily and constantly changing schedules, waking up on time for class, and being a self-advocate. Struggle with ability to resist distractions and temptations to meet goals. | Support reflective thinking and prompt ability to plan and carry out goals. Collaborative process of goal identification and plan development. Identify academic plan with concrete steps, reflect on previous academic history and current interests and goals, and provide unconditional support toward self-determination. | Barkley |
| Ahmann & Saviet 2019 | Quantitative | Brain disorder characterized by symptoms of inattention (difficulty staying focused) and/or hyperactivity-impulsivity affecting an individual's functioning and/or development. | Collaborative, supportive, goal-oriented - identify goals, develop self-awareness, systems, skills, and strategies necessary to achieve full potential | Barkley |
| Saviet & Ahmann 2020 | Qualitative | Neuropsychological disorder of EF characterised by symptoms of inattention (difficulty staying focused) and/or hyperactivity-impulsivity affecting functioning and/or development | Flexibility in accessibility and location helpful due to time and organisation issues. Telephone reduces distractions and encourages rapid rapport. Combination of in-person and online may increase motivation. | Barkley,  Brown |

| **Table 3: Analysis of Other Treatments** | | | | |
| --- | --- | --- | --- | --- |
| **Author/Date** | **Intervention Style** | **Characterisation** | **Aims/Outcomes** | **Theory** |
| Hecker et al 2002 | Individual | EF deficits; difficulty sustaining attention, leads to more off- task behaviour, minimising acquisition of academic information. | Reductions in effort to maintain attention should lower stress and fatigue associated with reading, increasing time spent reading | DSM IV |
| McMurray 2004 | Individual | Deficits in behavioural inhibition and sustained attention | Help synchronize brain activity and improve ability to maintain attention and cognitive focus. | Barkley |
| Fox et al 2005 | Guidance | Inattention, hyperactivity, impulsivity. Executive Function - dopaminergic networks. Central Nervous dysfunction - maturational lag or cortical under arousal | Improve attention, reduce impulsivity, control hyperactive behaviours, and increase cortical arousal. Produce long-term change using operant conditioning techniques to reinforce electrophysiological activity. | DSM IV TR |
| Loo & Barkley 2005 | Guidance | Maturational delay marked by under arousal | Critique – Address methodological problems in outcome research to demonstrate real treatment effect. Systematically monitor and report side effects. Diagnostic utility appears promising but further research is needed. | Barkley |
| Rybak et al 2006 | Individual | Deficits in EF; mood-independent circadian phase delay significantly impacts core pathology; disorder of arousal, sleep, affect and attention or vigilance. Neurobiological similarities to sleep disorders | Improve core subjective symptoms and/or neuropsychological functioning: alleviate depressive symptoms and cause a phase advance in daily sleep and activity rhythms | Barkley, DSM IV |
| Rybak et al 2007 |  | Affective disturbances and deficits in arousal and EF. Chronobiologic disturbances may also contribute to the pathophysiology and disability | Quantify seasonal mood change and circadian preference and assess how chronobiologic factors relate to subjective and neuropsychological functioning | Barkley, Brown |
| Fisher 2007 | Individual | Difficulty regulating emotions. Brain is hyper aroused | Enhances affect regulation and optimises brain plasticity and brain function through operant conditioning of firing frequencies | No primary citation |
| Ramsay 2010 | Guidance | Developmental syndrome - impairment may not arise until normative environmental demands exceed EF capabilities. Capacity for behavioural inhibition and foresight foundational for self-control and regulation. Developmentally inappropriate impulsivity and overactivity coincides with attention and information processing difficulties. | Critique -More examples of quality studies needed to establish as conventional treatment and assessment option. Working memory findings promising but initial and limited. | Barkley, Douglas, Nigg |
| Virta et al 2010 | Individual | EF processing, emotion modulation, and memory utilisation; hyperactivity, impulsivity, inattention. Negative cognitions and beliefs result in self-esteem problems | Feasibility and efficacy of hypnotherapy  and impact on symptoms, mood, quality of life, and cognitive performance | DSM IV, Brown |
| Gapin et al 2011 | Guidance | Deficits in EF; inattention, hyperactivity, and/or impulsivity; working memory, response inhibition, and planning; | Positive impact of physical exercise (PE) on cognitive abilities | DSM IV TR, Barkley |
| Halperin & Healey 2011 | Guidance | Chronic, highly prevalent neurodevelopmental disorder; Executive function deficits | Directed play and PE to promote neural and cognitive growth that would serve to reduce symptom severity across development. | DSM IV, Barkley, Douglas, Sergeant, Sonuga-Barke, Sagvolden |
| Archer & Kostrzewa 2012 | Guidance | Emotional self-regulation deficits, executive functioning problems. Emotional impulsiveness difficulties with delay of gratification | Reduce stress and negative affect, anxiety, depression, and self-destructive behaviour, improve acceptable behaviour, and cognitive function | DSM IV, Barkley |
| Mayer et al 2012 | Individual | Inattentive and hyperactive-impulsive; Executive function issues - attention, impulsivity, working memory | Improve core symptoms and mood | Sergeant, Barkley |
| Peterson 2012 | Guidance | Decreased beta wave states linked to lack of focus or difficulty maintaining attention towards tasks | Ways to produce beta-wave brain states to improve ability to focus and maintain attention | No primary citation |
| Bidwell et al 2012 | Guidance | Developmentally inappropriate levels of inattention, hyperactivity, and impulsivity. Executive function deficits; weaknesses in multiple cognitive domains | Identify processes that underlie clinical impairment and most promising neuropharmacological targets to alter those processes | DSM IV TR, Barkley, Sonuga-Barke, Sergeant |
| Stern et al 2012 | Individual | EFs include response inhibition, initiation, implementing strategies for performance, shifting, intrusion control, working memory and control of complex cognitive or motor responses | Focus on working memory, inhibition, shifting/dividing attention and persistence. | DSM IV, Barkley, Brown, Wender |
| Irvine 2013 | Individual | Executive function - organisation, planning, project management | Increase self-monitoring to reduce time perception discrepancies and increase treatment fidelity and compliance | Barkley |
| Abramovitch et al 2013 | Qualitative | neuro- developmental disorder characterized under-activity in prefrontal/striatal brain, and dopaminergic connectivity deficiencies | PE questionnaire analysis comparing physical activity to prevalence of anxious thoughts. | DSM IV TR, Barkley |
| Fuermaier et al 2014 | Individual | Executive dysfunctions and attention; working memory, inhibitory control, distractibility, vigilance, set-shifting, and task planning | Improve attention and cognitive functioning | Barkley |
| Gropper et al 2014 | Individual | Deficits in processing speed and executive functions, especially working memory | Seeking cognitive improvement, skill generalisation and transfer and functioning gain persistence. | DSM IV |
| Hiltunen et al 2014 | Individual | Emotional dysregulation; Executive Function processing - processing speed, regulating alertness, organising and prioritising, and utilizing memory; hyperactivity, impulsivity, inattention. | Further evaluate improvement maintenance and difference in efficacy between CBT and hypnotherapy at six-month follow-up. | DSM IV, Brown |
| Mawjee et al 2015 | Individual | Working memory impairments. | Intensive training will increase working memory capacity | DSM IV, Barkley |
| Gapin et al 2015 | Individual | Inappropriate levels of inattention and/or hyperactivity. Deficits in cognitive functioning: EF including self-monitoring and/or self-regulating | Impact of acute exercise on cognitive functions, and explore influence on BDNF | DSM IV TR, Nigg |
| Cosmo et al 2015 | Individual | EF deficits such as inhibitory control and attention | Increase cortical connectivity to improve EF such as inhibitory control and attention. | DSM IV TR |
| Salomone et al 2015 | Individual | attention, impulsivity, and overactivity. Impairments in higher-level executive functions such as inhibition and attention | Use cognitive rehabilitation principles to increase arousal to aid control of sustained attention | Barkley |
| Den Heijer et al 2016 | Systematic review | Inattention, impulsivity, impaired inhibition, and hyperactivity; Impaired executive functions | Overview on effect on cognitive, behavioural/ socio-emotional, and physical/ neurophysiological outcomes | DSM V, Barkley |
| Cowley et al 2016 | Individual | Neurobiological condition; self-regulation and executive functions. under- arousal in the cortical systems with excess slow wave activity affecting information processing | Train self-regulation of power in specific bands of the EEG frequency spectrum, through operant conditioning | Brown, Nigg, Sergeant,  Sonuga-Barke |
| Coogan et al 2016 | Guidance | Behavioural and attentional problems and sleep disturbance – circadian timekeeping altered and rhythm abnormalities implicated | Recommends light therapy and behavioural interventions to manage evening chronotype preference and late melatonin onset | No primary citation |
| Stern et al 2016 | Individual | Developmental impairment -deficient EF -regulatory processes for selecting, initiating, implementing, and overseeing thought, emotion, behaviour, and certain facets of motor and sensory functions. | Repetitive cognitive training over a circumscribed time frame to enhance cognitive abilities and skills. | Brown, Barkley, Nigg |
| Mayer et al 2016 | Individual | Neurodevelopmental disorder; difficulties in resource provision for attention processes and regulation of excitability thresholds. EF deficits in inattention-disorganisation | Improve activation and deactivation of the brain by regulation of cortical excitation and inhibition | DSM V, Nigg |
| Barth et al 2017 | Individual | Neuropsychiatric disorder - inattention, elevated levels of rest- lessness as well as impulsivity | Neurofeedback - Operant conditioning with reinforcement of desired activation patterns. | DSM V |
| Coogan & McGowan 2017 | Systematic review | Attention difficulties, impulsivity and hyperactivity; sleep disturbance | Core symptoms can be contributed to by poor sleep quality or quantity - sleep disturbance may be important in the aetiology of ADHD. | DSM V |
| Mancera et al 2017 | Individual | Neurobiological and neuropsychological disorder characterized by inattention, hyperactivity, and impulsivity. | Train cognitive abilities to increase motivation and to support the learning process. | No primary citation |
| Mawjee et al 2017 | Individual | Working memory impairments. | Strengthening working memory by repeated use – comparing effectiveness of two training lengths versus control | DSM IV, Barkley |
| Scheithauer et al 2017 | Individual | Inattention, hyperactivity, and impulsivity | Self-monitoring: teaching observation and recording of behaviour aiming to change the behaviour in the future. | DSM IV, DSM V |
| Schonenberg et al 2017 | Individual | Mental illness: marked difficulties to sustain attention, enhanced distractibility, impulsive, and hyperactive behaviour; emotional instability | Deviant brain activity patterns voluntarily modulated by operant learning strategies; Normalisation of brain activity will translate into improved cognitive and behavioural functioning | DSM IV TR, Wender |
| Allenby et al 2018 | Individual | Disease characterized by symptoms of impulsivity, inattention, and hyper- activity | Activation of cognitive control network with concurrent training task would improve performance of cognitive control and impulsivity | Barkley, Quay |
| Lacount et al 2018 | Guidance | Deficits in executive functioning - working memory, response inhibition, and organization; hypodopaminergic state in the prefrontal cortex | Neurobiological effects of PE are possible mediators of neurotransmitters and BDNF | DSM V, Barkley, Nigg, Sonuga-Barke |
| Mirzaiyan et al 2018 | Literature review | Neuropsychiatric disorder; deficits in executive functions with abnormal levels of impulsivity, inattention, and hyperactivity | Review of tDCS efficacy and mechanisms. Dysregulation of brain cortex excitability may result in the emergence of pathologic symptoms | DSM IV |
| Paz et al 2018 | Individual | Common neurobehavioral disorder where clinical manifestations are suspected to evolve from difficulties in attention and executive functions | possible beneficial effect of both right and left prefrontal activation on cognitive functions and deleterious effect on mood of right hemispheric stimulation | DSM V |
| Schindler 2019 | Individual | Attention, concentration, and memory, focus; Deficits in higher level cognitive skills – executive functioning, ability to manage time, prioritize assignments, and break large assignments into subcomponents. | Assist students in developing the academic, social, and psychological skills to succeed in college with 1-1 peer mentoring support | DSM V |
| Kallweit et al 2019 | Individual | Delay Aversion - Compared with healthy persons, those with ADHD may need higher and more salient reinforcement to improve their performance or perform like matched controls | Assess influence of physical activation, stimulation, and reward on cognitive performance and subjective symptoms | DSM IV, Barkley, Sagvolden, Sergeant, Sonuga-Barke |
| Mehren et al 2019 | Individual | Impairments in executive function: difficulties to sustain attention, increased distractibility, or reduced control of response interference | Investigate acute effects of aerobic exercise on EF and associated brain activation | DSM V,  Barkley |
| Mehren et al 2019 | Individual | Response inhibition; Hyperactivity, impulsivity, inattention core symptoms | Enhance response inhibition performance and task-related brain activation to improve cognitive functioning. | DSM V, Barkley |
| Rassovsky & Alfassi 2019 | Individual | Deficit in EF - sustained and divided attention, attentional and inhibitory control, working memory, planning, cognitive flexibility, and problem solving. | Examine influence of physical exercise during performance of an attentional task | DSM V |
| Dieber et al 2020 | Individual | Inattention and impulsivity/hyperactivity; diminished perception of sensory stimuli, and internally oriented state favouring mind wandering and attentional lapse; response inhibition | Reduce alpha power to explore short-term plastic effects on omission (i.e. perceptual) and commission (i.e. motor inhibition) error | DSM IV |
| Korman et al 2020 | Literature review | Attention, EF deficits: planning, inhibition and set-shifting, motor functioning, skill learning, emotional instability, sleep problems. Unstable and low arousal results in inability or difficulty to sustain attention | Improve core symptomology, compensate for adverse effects of medication, and augment or prevent psychiatric comorbidities | Douglas |

| **Table 4: Analysis of Psychotherapeutic Treatments** | | | | |
| --- | --- | --- | --- | --- |
| **Author/Date** | **Intervention Style** | **Characterisation** | **Aims/Outcomes** | **Theory** |
| Bemporad & Zambenedetti 1996 | Individual | Organisation and concentration difficulties, impulsivity, emotional lability. Difficulty estimating activity time; settling down to work; being free of distractions. Stimulus or excitement seeking influences behaviours and leads to negative outcomes in all domains, particularly relationships | Psychoeducation followed by analysis of detrimental defences/resistances constructed automatically over time, and alteration of negative core sense of self. Transferential distortions of happy/cheerful or naughty child need to be forgiven - problem solving approach to reconstruct childhood. | Wender |
| Gilmore 2000 | Individual | Difficulty regulating behaviour and affective processes, hypersensitivity to and poor modulation of stimuli, craving for sensory input, and intrusion of drive derivatives that disrupt coherent narrative. Disturbance in synthetic, organizing, and integrative function of the ego. Regulation, modulation, and balancing of internal and external stimuli; ego functions recruited for conflict and defence - contribute to fantasy | Organize self-states; integrate transference distortions into coherent, historically meaningful narrative; synthesize appearance of defence with dissociated affects and drive derivatives - both transparent and rigidly disavowed | DSM IV |
| Bemporad 2001 | Individual | Personality style magnifies neurologically based tendency to “escape into action” into systematic denial of any unpleasant circumstance by diverting attention to less threatening subjects or activities. Transference distortions of pain or confrontation avoidance by becoming cheerfully diverting or minimising irresponsible or irritating behaviour by acting as remorseful child. Absence of empathy due to preoccupation | Multimodal approach including psychoeducation (lifeskills or cognitive rehabilitation) Psychotherapy to correct erroneous views of self and others, awareness of defences and resistance arising from psychological pain | Wender |
| Carney 2002 | Individual | Inattention, impulsivity, hyperactivity, Response inhibition, inability to delay response, self-regulation, executive function deficits | Behaviours defensive and compensatory. Assist with cortical maturation via neuroplasticity to develop self-regulation and empathy | Barkley |
| Levin 2002 | Guidance | Crave stimulation, avoid boredom, needs for stimulation, tactile and visual nearness, self-control, and minimal delay | Recommends eclectic interdisciplinary approach to include psychoanalysis and cognitive remediation | No primary citation |
| Rafalovitch 2002 | Literature review | Result of neurochemical processes in the brain. Fragmented ego - unpredictable relationship between ego and the world, influenced by family dynamics. Behaviours are a mechanism of survival in response to anxiety around inability to function in environments of daily living. | Awareness of behaviour assists therapeutic work - syndrome with a complex of symptoms rather than a disorder with a specific set of symptoms | DSM IV, Wender, Barkley |
| Rothstein 2002 | Individual | Difficulties with tasks that require efficient execution of mechanical procedures and problem solving. Behavioural features: inconsistencies in attention, hypersensitivity to noise or other extraneous stimuli, excessive thought diversions, hyperactivity, drivenness, impulsivity, and fidgetiness. Compromised ability to learn. | Recognise internal experience of inattention, hyperactivity and impulsive states, incorporation in unconscious fantasies, and employment in service of both self-punitive urges and defences against the “unpleasure” associated with psychic conflicts | DSM IV |
| Zabarenko 2002 | Individual | EF network issues: style of ego functioning with impaired defensive and transferential capacities. Developmental impairments in capacity to make use-dependant modifications to brain circuitry. Deficits in visual memory and ability to categorise - pervasive impulsivity, cognitive control, and attention issues | Highly situation dependant - capacities are outgrowth of synthetic function of ego. Disturbed process of integration and internalisation that benefits from treatment but may be limited in modification | Barkley, DSM IV |
| Wright 2006 | Individual | Idiopathic, heterogeneous syndrome - disturbed attentional processes, impulsiveness, poor self-regulation, and impaired motor control. Dysfunction in EF - impairments in response inhibition, resistance to distraction, delay of gratification, sequencing, working memory, and regulation of attention and arousal. Impoverished model of self as agent. Disrupted insight into the meaning and intent of behaviour | Recommends reconceptualising disorder as impoverished development of a relatively stable, working representational model of an executive self-as-agent. | Barkley |
| Zabarenko 2011 | Guidance | Neuropsychiatric disorders. Executive disfunction disorder with pervasive impulsivity, visual memory deficits, working memory, goal neglect, and mind wandering in an executive-control task | Organic nature of the illness can make patients unsuited for the strenuous work of self-discovery. Think of statistical tools as iterative; Use clinical intuition to conceptualize data | Barkley, DSM IV |
| Conway 2012 | Systematic review | Deficits in executive brain function correlate directly with deficits in ego functions. Concept of time not being fully constructed leads to improper cathexis to an object - difficulties with "reality sampling". reality can be experienced by the child’s ego as an overwhelming source of stimulation Alternatively, ADHD is a “syndrome” determined by the quality of the parental relationship, underlying personality structure due to disturbances in early object relations—trauma, ego- and self-development, and interpersonal challenges from family life. | Disruptions in the early attachment relations may be due to trauma. Unresolved interpersonal conflicts arising from disturbances in object relations can lead to conflicting internal states. Use of the transference to intervene at the level of object relations is central to therapeutic change | Barkley |
| Waska 2014 | Individual | Symptoms are the result of a psychological conflict rather that a biological brain abnormality. Perceived as oppositional struggle to internal object. Internal struggle for control resulted in procrastination, resistance and avoidance behaviours. | Object detachment process resulting in overwhelm from unconscious relational conflicts. Symptoms part of an organised defence system that can be challenging to treat as there may be resistance to general exploration of areas of distress. | No primary citation |
| Conway 2015 | Literature review | Object relation shaped by disorganizing or traumatizing attachment relationships. Childhood trauma and insufficient attachment interfere with ability to mentalise, or experience empathy. This disrupts the ability to self-regulate (ego organisation) | Recommends integrated ego and relational framework focused on developing mentalisation abilities to strengthen ego functioning. Help remember past experiences in the framework of stable and empathic analytic relationship, and thereby rebuild the capacity for mentalization. Empathy development assists with self-reflection and self-regulation | Barkley, DSM V |
| de Almeida Silva 2016 | Evaluation | Inconsistency and variability in the integrative, organizing, and synthetic functions of the ego, narcissistic fragility and need to be in control. Notable phenomenological similarities between ADHD and narcissistic/borderline personality | Highlights lack of attainment of the reality principle and lack of appreciation of the separateness and constancy of those out- side the self can lead to more frequent use of immature and neurotic defense mechanisms related to difficulties in impulse control (acting out) and distortion in the representation of the self and others (projection, splitting, idealization, fantasy, denial, omnipotence, displacement). | Barkley, DSM IV TR, Wender |
| Shaikh 2018 | Group | Problems with global and social self-esteem, as well as psychosocial competence, perspective-taking ability, the ability to heed, receive, and respond to social cues, and increased self-consciousness | Improve self-esteem and social development in college students by taking into account the whole person, including how individuals feel about themselves and others. Psychoeducation, goal setting, and group participation as “live laboratory” for skills development. | Barkley |
| Lusk 2019 | Individual | Inattention, impulsivity, and hyperactivity. Developmental difficulties such as affective dysregulation and memory issues. A symptom complex that can stem from a multiplicity of early-life determinants, some of which may be constitutional, and executive functioning disturbances. Equally important are psychological determinants, or the internalization of trauma and the developmental status. Social determinants include a wide variety of adverse early-life experiences. | Importance of using a multimodal and developmental approach. Holistically investigate unique individual expression and development in relation to neuropsychological dysfunction. Disturbances in ego functions by failure of the primary object to contain and center attention, and to construct in a co-established temporality seen in struggle with cohesive sense of self, impaired capacity for self-observation, and self-reflection. Helping understand and accept developmental limitations through careful analysis of defenses against shame is necessary. | Barkley, DSM V |
